# Kinetically Stabilized Cation Arrangement in Li_3_YCl_6_ Superionic Conductor during Solid‐State Reaction

**DOI:** 10.1002/advs.202101413

**Published:** 2021-06-17

**Authors:** Hiroaki Ito, Kazuki Shitara, Yongming Wang, Kotaro Fujii, Masatomo Yashima, Yosuke Goto, Chikako Moriyoshi, Nataly Carolina Rosero‐Navarro, Akira Miura, Kiyoharu Tadanaga

**Affiliations:** ^1^ Graduate School of Chemical Science and Engineering Hokkaido University Kita 13, Nishi 8 Sapporo Hokkaido 060‐8628 Japan; ^2^ Joint and Welding Research Institute Osaka University 11‐1 Mihogaoka Ibaraki Osaka 567‐0047 Japan; ^3^ Nanostructures Research Laboratory Japan Fine Ceramics Center 2‐4‐1, Mutsuno, Atsuta, Nagoya Aichi 456‐8587 Japan; ^4^ Creative Research Institution Hokkaido University Kita 21, Nishi 10 Sapporo Hokkaido 001‐0021 Japan; ^5^ Department of Chemistry, School of Science Tokyo Institute of Technology 2‐12‐1 W4‐17 O‐okayama, Meguro Tokyo 152‐8551 Japan; ^6^ Department of Physics Tokyo Metropolitan University 1‐1 Minami‐Osawa Hachioji Tokyo 192‐0397 Japan; ^7^ Graduate School of Advanced Science and Engineering Hiroshima University 1‐3‐1 Kagamiyama Higashihiroshima Hiroshima 739‐8526 Japan; ^8^ Faculty of Engineering Hokkaido University Kita 13, Nishi 8 Sapporo Hokkaido 060‐8628 Japan

**Keywords:** density functional theory, halides, in situ XRD, neutron diffraction, solid electrolytes

## Abstract

The main approach for exploring metastable materials is via trial‐and‐error synthesis, and there is limited understanding of how metastable materials are kinetically stabilized. In this study, a metastable phase superionic conductor, *β*‐Li_3_YCl_6_, is discovered through in situ X‐ray diffraction after heating a mixture of LiCl and YCl_3_ powders. While Cl^−^ arrangement is represented as a hexagonal close packed structure in both metastable *β*‐Li_3_YCl_6_ synthesized below 600 K and stable *α*‐Li_3_YCl_6_ above 600 K, the arrangement of Li^+^ and Y^3+^ in *β*‐Li_3_YCl_6_ determined by neutron diffraction brought about the cell with a 1/√3 *a*‐axis and a similar *c*‐axis of stable *α*‐Li_3_YCl_6_. Higher Li^+^ ion conductivity and lower activation energy for Li^+^ transport are observed in comparison with *α*‐Li_3_YCl_6_. The computationally calculated low migration barrier of Li^+^ supports the low activation energy for Li^+^ conduction, and the calculated high migration barrier of Y^3+^ kinetically stabilizes this metastable phase by impeding phase transformation to *α*‐Li_3_YCl_6_. This work shows that the combination of in situ observation of solid‐state reactions and computation of the migration energy can facilitate the comprehension of the solid‐state reactions allowing kinetic stabilization of metastable materials, and can enable the discovery of new metastable materials in a short time.

## Graphical abstract

A metastable superionic conductor, *β*‐Li_3_YCl_6_, is synthesized by heating a mixture of LiCl and YCl_3_ powders. The computationally calculated low migration barrier of Li^+^ supports the low activation energy for Li^+^ conduction, and the calculated high migration barrier of Y^3+^ kinetically stabilizes this metastable phase by impeding phase transformation to thermodynamically stable *α*‐Li_3_YCl_6_.

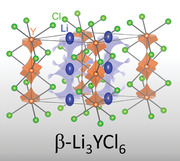

## 1 Introduction

The number of materials that are experimentally synthesized by solid‐state reactions has gradually increased, while the number of computationally predicted materials, especially thermodynamically metastable materials, has rapidly increased.^[^ ^1^, ^2^, ^3^, ^4^, ^5^, ^6^ ^]^ The synthesis of metastable materials and understanding how these metastable materials are kinetically stabilized are the heart of material chemistry for further exploring new materials.^[^ ^6^ ^]^ Recent in situ characterization studies have revealed that solid‐state reactions using powder reactants often evolve via a variety of intermediates,^[^ ^7^, ^8^, ^9^, ^10^, ^11^, ^12^, ^13^, ^14^ ^]^ some of which are characterized as new materials formed under nonequilibrium conditions.^[^ ^11^, ^12^ ^]^ However, a general understanding of solid‐state reactions under nonequilibrium conditions is still lacking. In particular, when the thermodynamic driving forces of multiple reactions are small and compete, the reaction of solids is dominated by kinetics.^[^ ^13^, ^14^ ^]^ Metastable phases are formed during synthesis when the kinetic barrier impedes the reaction before equilibrium is reached.^[^ ^6^, ^7^, ^15^ ^]^ Because the migration barrier of each atom in solids can be determined computationally,^[^ ^16^, ^17^, ^18^ ^]^ this energy barrier can potentially be exploited for evaluating the synthesizability of computationally predicted compounds.

Li^+^‐ion conducting electrolytes are key materials for all‐solid‐state batteries, which are attractive next‐generation batteries with high safety and high energy density. Various oxides, sulfides, halides, and hydride solid electrolytes have been developed.^[^ ^19^, ^20^, ^21^ ^]^ Halide‐based electrolytes have been studied for several decades; however, except for the high‐temperature phase of Li_3_InBr_6_, their conductivities had been considered to be rather small at room temperature.^[^ ^22^ ^]^ After the discovery of the high Li^+^ conductivity of Li_3_YCl_6_ and Li_3_YBr_6_, i.e., 10^−4^–10^−3^ S cm^−1^ at room temperature, and their capability as solid electrolytes for all‐solid‐state batteries reported by Asano et al.,^[^ ^23^ ^]^ many ternary halides have been (re)investigated as solid electrolytes,^[^ ^24^, ^25^, ^26^, ^27^, ^28^, ^29^, ^30^, ^31^ ^]^ some of which have shown a conductivity of 10^−4^–10^−3^ S cm^−1^. The highly conductive features of Li^+^ in chlorides, bromides, and iodides can be understood as large and monovalent halide ions with weak interactions with Li^+^, which facilitates fast Li^+^ diffusion. Moreover, their wide electrochemical stability window and good stability toward oxide cathodes are advantageous.^[^ ^26^ ^]^

Ternary chlorides are formally represented as Li_3_MX_6_ (M = rare earth metal of La‐Lu, Sc, Y; X = Cl, Br) composed of closely packed structures of X^−^ and different cation occupancies in octahedral holes.^[^ ^26^ ^]^ For instance, Li_3_YCl_6_ is composed of hexagonal close‐packed Cl^−^, whereas Li_3_YBr_6_ is composed of cubic close‐packed Br^−^.^[^ ^23^ ^]^ Cation arrangements depend on the composition and synthesis method.^[^ ^23^, ^29^, ^30^ ^]^ Considering that different cation arrangements of Li_3_ErCl_6_ show comparable calculated energies,^[^ ^29^ ^]^ the cation arrangements of these halides would be rather flexible. Indeed, various average structures as well as local structures have been reported, even for the same composition, which depends on the synthesis methods and heating temperatures.^[^ ^29^, ^32^, ^33^ ^]^ The effect of the Li^+^ concentration,^[^ ^27^ ^]^ the blocking effect of rare earth elements,^[^ ^27^, ^29^ ^]^ the disordering of M atoms,^[^ ^29^ ^]^ and the volume and distortion of MCl_6_^3−[^ ^29^, ^34^ ^]^ on Li^+^ conductivities have been discussed. Because these cation sublattices are correlated with ion conductivities, the synthesis strategy for controlling the cation arrangement paves the way for exploring new solid electrolytes.

Metastable ion conductors such as Li_7_P_7_S_11_ and LiAlCl_4_ formed via solid‐state synthesis^[^ ^35^, ^36^, ^37^ ^]^ provide a prospectively interesting motif for understanding the accessibility to the metastable phase by exploring the mobility of each atom. Assuming that the fast transport of charge carriers does not govern the phase transition from the metastable to stable phase, the effect of the mobility of the constituent elements, excepting charge carriers, on the phase transition can be discussed. Herein, a new metastable phase of Li_3_YCl_6_ with a smaller lattice and high Li^+^ conductivity was discovered through in situ X‐ray diffraction (XRD) analysis of the solid‐state reaction between LiCl and YCl_3_. Structural analysis using X‐ray and neutron diffraction studies suggests that disordered Y^3+^ arrangement enhances the Li^+^ conductivity, whereas ab initio studies show that the large migration barrier of Y^3+^ kinetically stabilizes this metastable Li_3_YCl_6_.

## 2 Results

**Figure 1:**
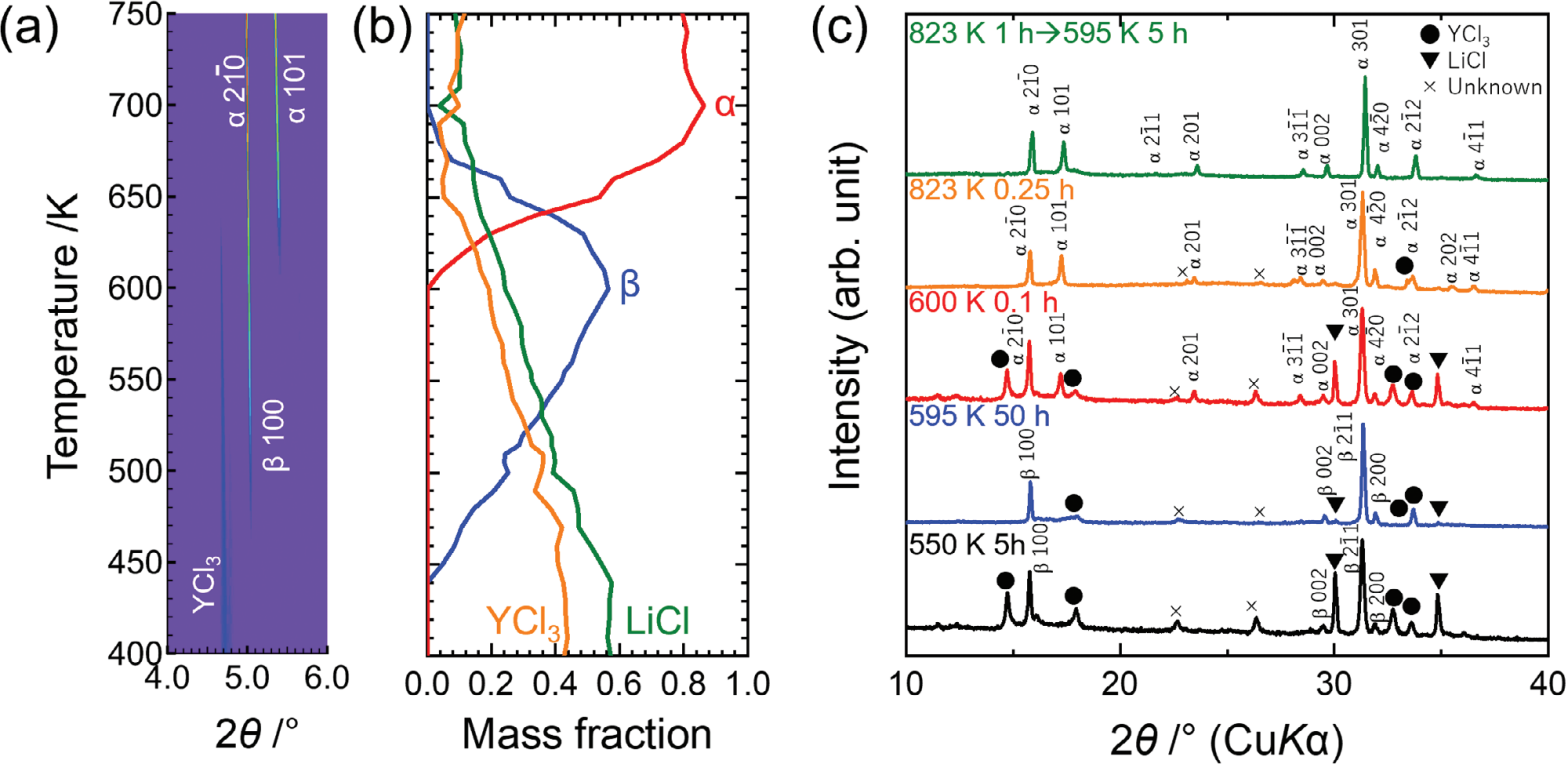
Solid‐state reaction between LiCl and YCl_3_ for the synthesis of Li_3_YCl_6_: a) temperature dependence of synchrotron X‐ray diffraction and b) mass fraction calculated from Rietveld refinement results. The wavelength of incident X‐ray was 0.496391 Å. c) XRD patterns of Li_3_YCl_6_ synthesized at different temperatures and heating times measured using CuK*α* radiation (*λ* = 1.5418 Å).

In situ XRD upon heating the mixture of LiCl and YCl_3_ at 30 K min^−1^, as shown in **Figure** **1**a, shows that the diffraction peak at ≈5.0° appeared above 450 K and an additional peak appeared at ≈5.4° above 600 K. Both peaks can be assigned to the reported hexagonal Li_3_YCl_6_;^[^ ^23^ ^]^ however, the absence of the peak at ≈5.4° in the temperature range of 450–600 K indicates the formation of a new phase with higher symmetry and/or smaller lattice parameters. Hereafter, previously reported phase and this new phase are referred as *α*‐Li_3_YCl_6_ and *β*‐Li_3_YCl_6_, respectively. The new phase, *β*‐Li_3_YCl_6_, can be assigned as the cell with a 1/√3 *a*‐axis and a similar *c*‐axis of reported *α*‐Li_3_YCl_6_. Thus, the reaction can be formulated as follows, and their mass fraction using the structural model described later is shown in Figure 1b (Figure S1, Supporting Information)

(1) $$3\mathrm{LiCl}+\mathrm{YC}l_{3}\to \beta -Li_{3}\mathrm{YC}l_{6}$$

(2) $$\beta -Li_{3}\mathrm{YC}l_{6}\to \alpha -Li_{3}\mathrm{YC}l_{6}$$

The *β*‐Li_3_YCl_6_ sample synthesized by heating a mixture of LiCl and YCl_3_ at 595 K for 50 h produced a nearly single‐phase powder (Figure 1c), which was used for structural and impedance measurements, as described later. There were two unindexed peaks. No electron diffraction spots corresponding to these unindexed peaks were seen in the electron diffraction image (Figure S2, Supporting Information), indicating no superlattice structures of *β*‐Li_3_YCl_6_. Intensities of these unindexed peaks increased when the sample was exposed to air for ≈1 min before heating (Figure S3, Supporting Information). Although these diffractions cannot be assigned as oxides, chlorides, and oxychlorides recorded in ICSD database, these peaks are likely attributed to an unknown impurity phase composed of Li^+^, Y^3+^, O^2−^, OH^−^, CO_3_^−^, Cl^−^, or H_2_O. A decrease in temperature remained in the unreacted starting materials, whereas a further increase in temperature at above 600 K yielded an *α*‐Li_3_YCl_6_ phase even after heating for only 15 min. At 595 K, the heat duration from 5 to 500 h showed *β*‐Li_3_YCl_6_ and did not bring about significant changes (Figure S4, Supporting Information). Furthermore, the synthesis by heating to 823 K and then annealing at 595 K for 50 h did not yield a *β*‐Li_3_YCl_6_; thus, the phase transition from *β*‐Li_3_YCl_6_ to *α*‐Li_3_YCl_6_ was irreversible, and *β*‐Li_3_YCl_6_ was metastable. As reported, the ball milling of LiCl and YCl_3_ together and a subsequent heating did not produce *β*‐Li_3_YCl_6_.^[^ ^23^, ^34^ ^]^ YCl_3_ composed of hexagonal close‐packed anion arrangement as that of Li_3_YCl_6_ but only one‐third octahedral site is filled with Y. Thus, we consider that a topotactic reaction occurs by diffusing Li and Cl from LiCl into YCl_3_ even below 600 K, leading to the formation of metastable *β*‐Li_3_YCl_6_ that cannot be obtained through high‐temperature heating or ball milling treatment.

**Figure 2:**
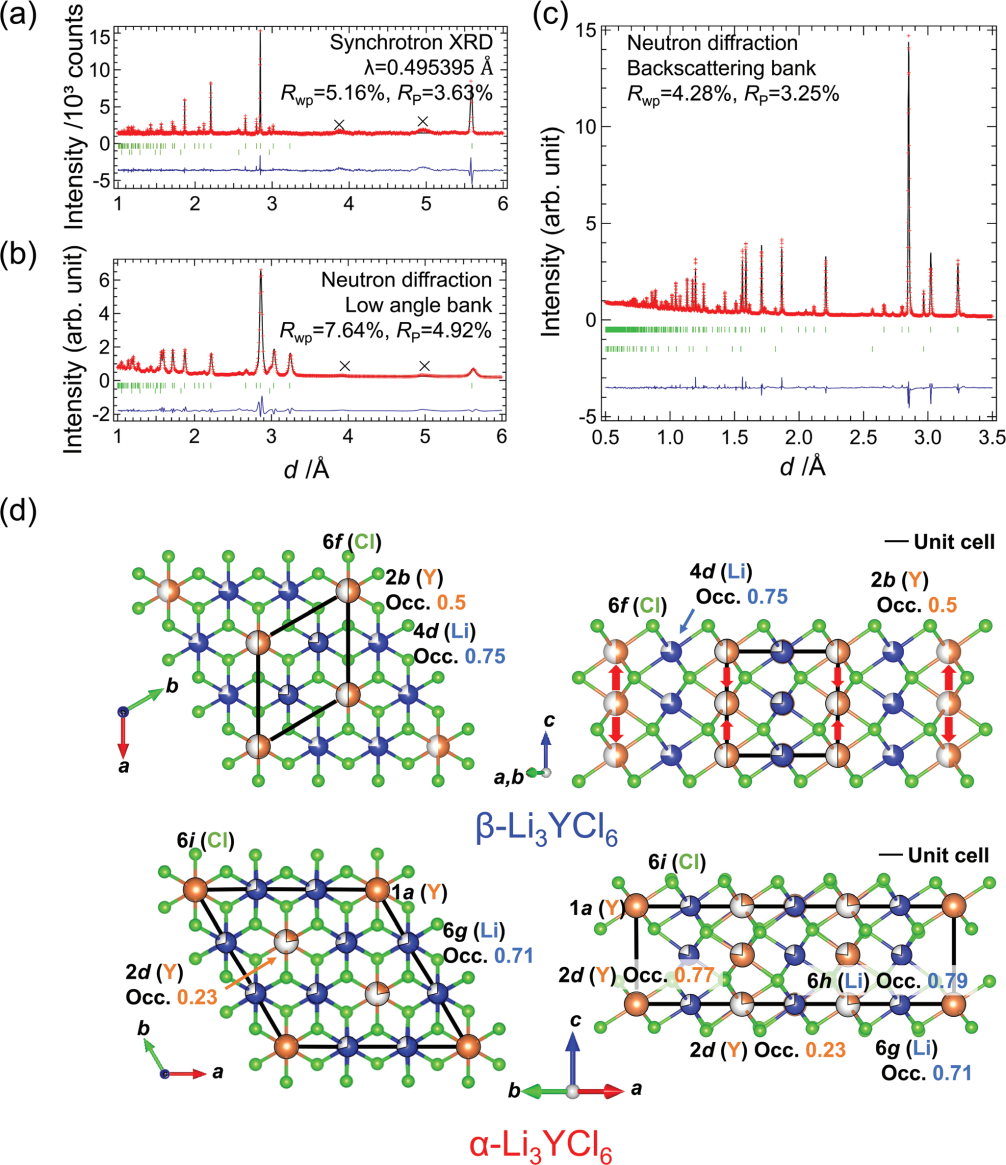
Rietveld profiles of a) XRD and b,c) neutron diffraction data for *β*‐Li_3_YCl_6_ synthesized at 595 K. The measurements are conducted at room temperature. Cross marks denote unidentified small diffraction peaks likely from an unknown impurity phase. The experimental and calculated results are indicated by dots and solid lines, respectively. The green bars are diffraction position of *β*‐Li_3_YCl_6_ and LiCl. The bottom lines are the residuals. The refinement details are in supporting document. d) Crystal structure of *β*‐Li_3_YCl_6_ and *α*‐Li_3_YCl_6_.^[^ ^34^ ^]^ The unit cells are denoted through the black lines, and the migrations of Y during the phase transition from *α*‐Li_3_YCl_6_ to *β*‐Li_3_YCl_6_ are represented by red arrows.

**Figure** **2**a–c shows the Rietveld profiles of *β*‐Li_3_YCl_6_ obtained through SXRD and ND, respectively. The main peaks in SXRD are assigned to the hexagonal cell with lattice parameters of *a* = 6.4604(1) Å and *c* = 6.0302(1) Å. Both the *a*‐ and *c*‐axes are expanded (per the same number of atoms) when compared with the lattice parameters of *α*‐Li_3_YCl_6_ synthesized at 700 K (1/√3*a* = 6.45754(2) Å, *c* = 6.02606(9) Å [Table S1, Supporting Information]). Thus, the density of *β*‐Li_3_YCl_6_ is lower than that of *α*‐Li_3_YCl_6_. Rietveld refinement results of neutron diffraction data collected at backscattering bank are shown in **Table** **1**; the refinement results of SXRD and ND collected at low angle bank are summarized in Table S2 (Supporting Information). *β*‐Li_3_YCl_6_ was crystallized with the space group *P* $\bar{3}$/*m*1. Here, Cl^−^ is in a hexagonal close‐packed anion arrangement, and every cation is octahedrally coordinated. There are two cation sites, as shown in Figure 2d. One is the Wyckoff position 2*b*, where Y is statistically occupied at the edge of the lattice along the *c*‐axis. In comparison with *α*‐Li_3_YCl_6_, *β*‐Li_3_YCl_6_ would be less stable because more Y^3+^ statistically existing as second nearest atoms induce the electronic repulsion between Y^3+^: Pauling's third rule. The phase transition from *β*‐ to *α*‐Li_3_YCl_6_ can be understood by the migration of Y along the *c*‐axis, as shown in red arrows in Figure 2d. The other is the Wyckoff position 4d, where the Li occupation is 0.75. The off‐stoichiometric and antisite defects did not improve the Rietveld fitting. The bond length between Li (4d) and Cl (2.62871(4) Å) was longer than that in LiCl (2.5730(15) Å: ICSD No. 52235), leading to the bond valence sum slightly less than one (0.857). The atomic displacement parameter of Y site was smallest (*U* = 0.0133(2) Å^2^), larger in Cl (*U* = 0.02097(6) Å^2^), and the largest in Li (*U* = 0.0522(4) Å^2^), as expected. Larger atomic displacement parameter of Li^+^ along the *c*‐axis was proposed: *U*_11_ = *U*_22_ = 0.03710(6) Å^2^, *U*_33_ = 0.0858(13) Å^2^. This anisotropic displacement suggests the Li^+^ conduction path along the *c*‐axis; this is also supported by the bond valence sum map (Figure S5, Supporting Information). Comparing with *α*‐ and *β*‐Li_3_YCl_6_ phases, no significant difference in effective coordination numbers^[^ ^38^ ^]^ derived by average structure is determined by diffraction techniques.

**Table 1:** Fractional coordinates, occupancies, atomic displacement parameters, and bond valence sum of *β*‐Li_3_YCl_6_. Neutron diffraction collected at backscattering bank. Anisotropic atomic displacement parameters, *U*_11_ and *U*_33_, were used for Li. Lattice parameter: *a* = 6.464667(7) Å, *c* = 6.043934(14) Å, *R*_wp_ = 4.28%, *R*_P_ = 3.25%, *R*_e_ = 1.15%, *R*_B_(Li_3_YCl_6_) = 8.53%, *R*_F_(Li_3_YCl_6_) = 10.25%, Space group:165 *P* $\bar{3}$ *c*1, The second phase: 2.5 mass% of LiCl

			Atomic coordinates				
Atom	Wyckoff position	Occupancy	*x*	*y*	*z*	*U* [Å^2^]	BVS
Y	2*b*	0.5	0	0	0	0.0113(2)	3.206
Li	4*d*	0.75	1/3	2/3	0	0.0533(4)^a)^	0.853
Cl	6*f*	1	1/3	0	1/4	0.02097(6)	0.961

^a)^ *U*_11_(Li) = *U*_22_(Li) = 0.03710(6) Å^2^, *U*_33_(Li) = 0.0858(13) Å^2^.

**Figure 3:**
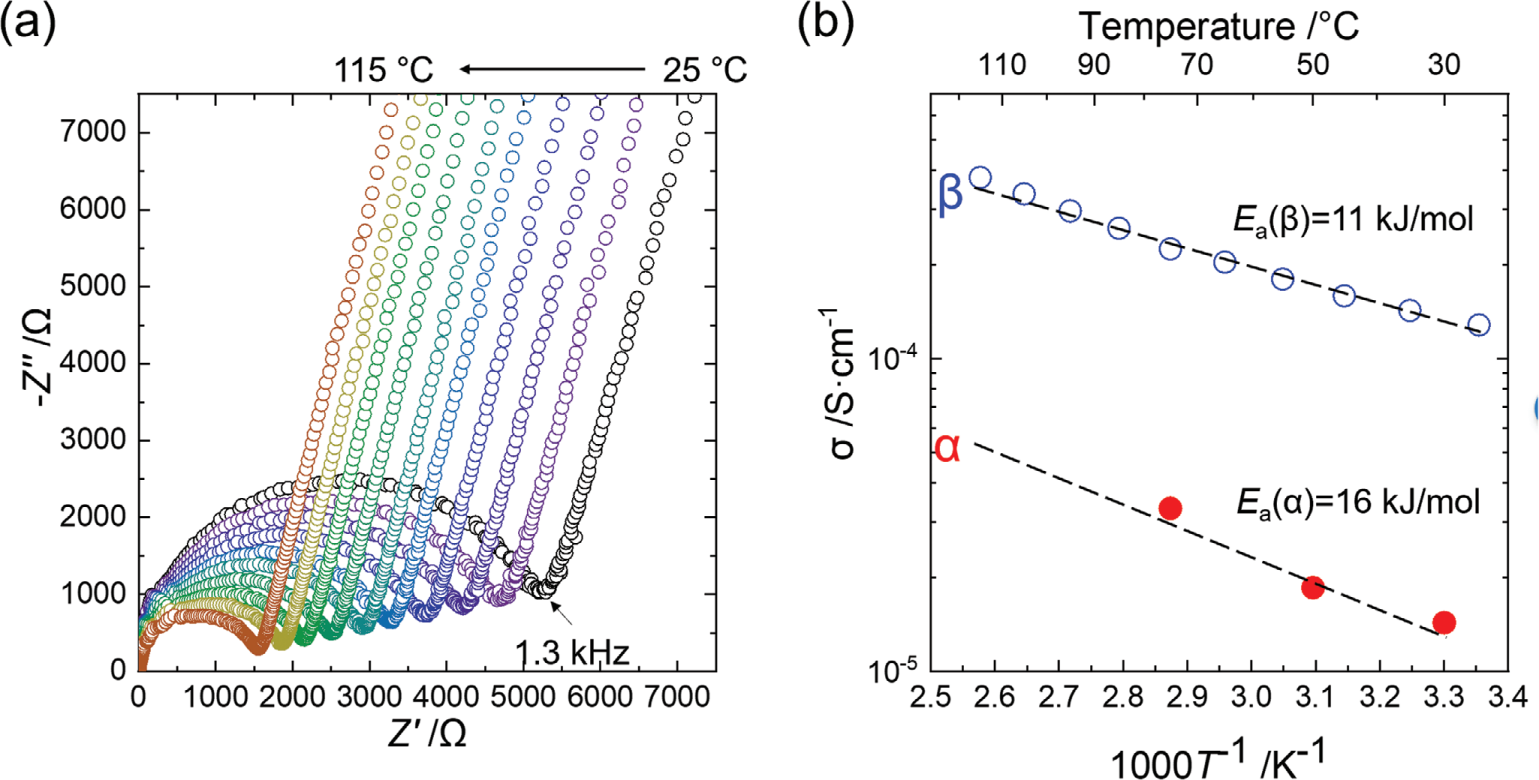
a) Nyquist plots of the AC impedance measurements of *β*‐Li_3_YCl_6_. b) Temperature dependence of Li^+^ conductivity in *α*‐ and *β*‐Li_3_YCl_6_ synthesized at 700 and 595 K, respectively. The values of the pre‐exponential factor in the Arrhenius equation were calculated as 4.31 for *β*‐Li_3_YCl_6_ synthesized at 595 K and 4.68 for *α*‐Li_3_YCl_6_ synthesized at 700 K.

**Figure** **3** shows the electrochemical impedance spectroscopy and temperature dependence of Li^+^ conductivity acquired through impedance measurements of cold‐pressed pellets of *α*‐ and *β*‐Li_3_YCl_6_ synthesized at 595 and 700 K, respectively. Li^+^ conductivity for *β*‐Li_3_YCl_6_ (1.2 × 10^−4^ S cm^−1^) at near room temperature is higher than that in the *α*‐Li_3_YCl_6_ (1.4 × 10^−5^ S cm^−1^), and their activation energies of *β*‐Li_3_YCl_6_ (11 kJ mol^−1^) are lower than that in the *α*‐Li_3_YCl_6_ (16 kJ mol^−1^). The values of the pre‐exponential factor of the Arrhenius equation, which are a product of the carrier concentrations and diffusion coefficients (see Equation (6) in the Appendix of the Supporting Information), are comparable. Although we cannot deny the possibility that these conductivities are limited by the grain boundaries, the crystallographic feature of the *β*‐Li_3_YCl_6_ can account for the high Li^+^ conductivity and low activation energy. The larger lattice parameters (per atoms) due to the repulsion between Y^3+^ widens Li^+^ pathway in *β*‐Li_3_YCl_6_, and thus lowers the activation energy and increases the conductivity of *β*‐Li_3_YCl_6_.

**Figure 4:**
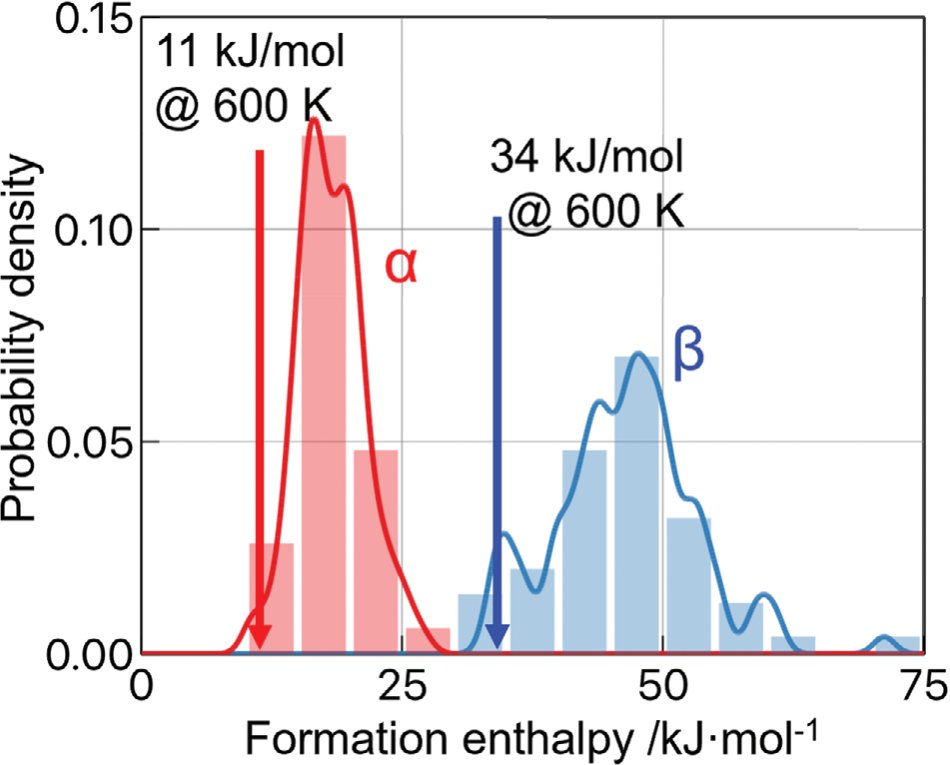
Calculated configurational density of states of *α*‐ and *β*‐Li_3_YCl_6_ by sampling 100 structures. The enthalpy of 3LiCl + YCl_3_ was set at zero.

Configurational density of states^[^ ^39^ ^]^ of *α*‐ and *β*‐Li_3_YCl_6_ is shown in **Figure** **4**. The microcanonical ensemble‐averaged formation enthalpies were evaluated as 11 and 34 kJ mol^−1^ (0.01 and 0.03 eV/atom) at 600 K for *α*‐ and *β*‐Li_3_YCl_6_, respectively. Though they are slightly positive, the free energies will be negative due to the contributions of finite temperatures including configurational entropy of atom. The small difference in these energies, 23 kJ mol^−1^ (0.02e V/atom), was experimentally supported by differential scanning calorimetry (DSC) measurements, which showed no clear heat signal during heating the *β*‐Li_3_YCl_6_ (Figure S6, Supporting Information).

**Figure 5:**
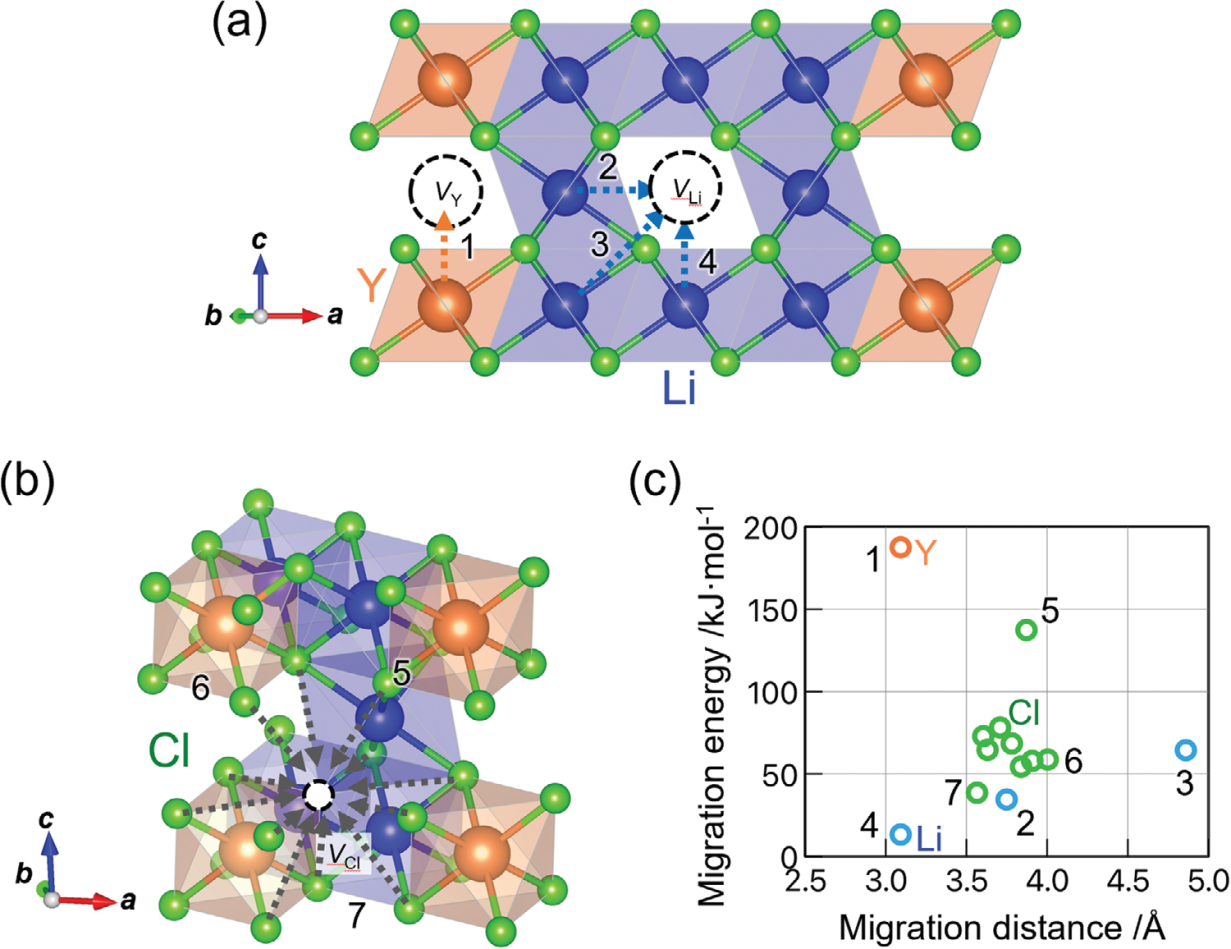
Computational migration path of a) Li^+^ (blue) and Y^3+^ (orange), and b) Cl^−^ (green) in *β*‐Li_3_YCl_6_. c) Migration energies as a function of migration distance. The numbers in the structures and the energy graph indicate each migration path. For Cl^−^, the paths with highest migration energy, longest *M*–*V* distance, and the lowest migration energy are numbered. *V*_Li_, *V*_Y_, and *V*_Cl_ represent the vacancies of Li, Y, and Cl, respectively.

**Figure** **5** shows that the migration energies of Li^+^, Cl^−^, and Y^3+^ were calculated as 13–63, 38–137, and 190 kJ mol^−1^, respectively. These energies are understandable based on their valence, ionic radii as well as atomic displacement presented in Table 1. The lowest migration energy of Li^+^ on the path between the nearest neighbor sites (13 kJ mol^−1^) is comparable to the experimental activation energy of the Li^+^ conductivity in the *β*‐Li_3_YCl_6_, as shown in Figure 3. The migration energy of Y^3+^ along the c‐axis was 190 kJ mol^−1^, which is significantly larger than that of Li^+^. The transition from the *β*‐ to the *α*‐ Li_3_YCl_6_ is locally considered to be due to the fact that approximately half of Y^3+^ migrates along the *c*‐axis (Figure 2d), together with the migration of highly mobile Li^+^. Assuming that the reaction proceeds via subsequent reactions proposed in Equations (1) and (2), the migration energy of Y^3+^ can be attributed to the activation energy of the phase transition, which is experimentally supported by the comparable activation energies derived from the temperature dependence of the phase fraction shown in Figure 1a, i.e., ≈180 kJ mol^−1^ (Figure S7, Supporting Information).

The temperature dependence of the jump frequency of Y^3+^ can be estimated from the Y^3+^ migration energy. The jump frequency of ions, *f*, is given by^[^ ^40^ ^]^

(3) $$f=f_{0}\;\exp\left(-\frac{E_{m}}{k_{B}T}\right)$$

where *f*_0_ and *E*_m_ are the jump trial frequency and migration energy of Y^3+^, respectively. The *E*_m_ was calculated to be 190 kJ mol^−1^, as described above. In this study, the jump trial frequency *f*_0_ was assumed to be 1 THz, which is the ordinary order for phonon vibrations of atoms based on first‐principles study.^[^ ^41^ ^]^ At 550 K, the jump frequency was calculated to be 2 × 10^−6^ Hz, which means that only 0.0002% of Y statistically migrates to the next site in a second. At 650 K, the value reached 0.001 Hz, suggesting 0.01% statistical migration in a second, which is ≈50 times higher than that at 550 K. Considering the phase transition from *β* to *α*‐Li_3_YCl_6_ at 600 K (Figure 1a), the less frequent migration of Y at lower temperatures also supports the kinetic stabilization of *β*‐Li_3_YCl_6_.

The reaction between LiBr and YBr_3_ to form Li_3_YBr_6_ was further investigated through in situ XRD measurements. Li_3_YBr_6_ was formed above 550 K, but the corresponding metastable phase was not detected (Figure S8, Supporting Information). Although the computationally determined energy difference between *β*‐Li_3_YBr_6_ and reported Li_3_YBr_6_ (13 kJ mol^−1^ @ 600K; Figure S9, Supporting Information) was slightly smaller than the energy difference of the corresponding chlorides described above (23 kJ mol^−1^ at 600 K; Figure 4), the calculated migration energy of Y^3+^ in *β*‐Li_3_YBr_6_ (140 kJ mol^−1^) was significantly smaller than that of yttrium in *β*‐Li_3_YCl_6_ (190 kJ mol^−1^). Therefore, this low migration barrier of Y^3+^ would not impede the reactions to kinetically stabilize *β*‐Li_3_YBr_6_, in contrast to *β*‐Li_3_YCl_6_.

## 3 Discussion

Notably, the *a*‐axis lattice parameter of *β*‐Li_3_YCl_6_ is 1/√3, which is the first example of a Li_3_MCl_6_ system. The low density of *β*‐Li_3_YCl_6_ due to large electronic repulsion between Y^3+^ can account for the low activation energy of Li^+^ transport and high conductivity. Comparison with the ionic conductivity of other reported *α*‐Li_3_YCl_6_ samples (Table S1, Supporting Information) shows that the conductivity of *β*‐Li_3_YCl_6_ was higher than that of reported *α*‐Li_3_YCl_6_, but was lower than that of Li_3_YCl_6_ synthesized by ball‐milling. The diffraction pattern of the ball‐milled Li_3_YCl_6_ can be indexed as *α*‐Li_3_YCl_6_, but the broad diffraction peak does not yield a detailed structural model.^[^ ^23^ ^]^ Nonetheless, partial disordering of Y is discussed because a broad and weak 101 diffraction peak was detected, which only appeared in the case of *α*‐Li_3_YCl_6_. A related study on the effect of Er disorder in ball‐milled Li_3_ErCl_6_ proposed that this disorder broadens the diffusion channel of Li;^[^ ^29^ ^]^ we can understand the enhancement of Li^+^ conduction in *β*‐Li_3_YCl_6_ with the disordered Y site in a similar way. However, the best cation arrangement for achieving high Li conductivity is still unclear. In this work, a new cation arrangement in Li_3_YCl_6_ with a different unit cell was discovered through the solid‐state reaction between LiCl and YCl_3_ powders, the conductivity of which is higher than that of *α*‐Li_3_YCl_6_ (Table S1, Supporting Information). Thus, in situ analyses of solid‐state reactions can facilitate the detection of various cation orderings, which would aid in the discovery of new ion conductors with high conductivity.

Calculations reveal that many ion conductors are metastable materials,^[^ ^35^, ^36^, ^37^ ^]^ and their synthesizability is thus an important issue. Sun et al. reported that higher average lattice cohesivities yield wider accessible energy ranges in which metastable compounds can be accessed,^[^ ^3^ ^]^ based on 29902 provenance‐filtered Materials Project entries.^[^ ^1^ ^]^ In this report, chlorides were found to exhibit stronger averaged cohesive energies and a wider accessible energy range in which metastable compounds can be accessed than bromides; this can explain the successful synthesis of the *β*‐phase only for Li_3_YCl_6_. Nonetheless, this thermodynamic understanding is not based on kinetics, and the effect of kinetics on each atom in each multicomponent material has not been described. Indeed, many computational studies on the kinetics of the conduction mechanism have recently attracted great interest, and thus have focused on mobile ions and their counter ions.^[^ ^17^, ^18^, ^42^, ^43^ ^]^ Nonetheless, for determining the synthesizability of computationally predicted ion conductors, not only mobile ions but also less mobile ions such as Y in Li_3_YCl_6_, should be considered to kinetically stabilize materials. When all atoms migrate sufficiently, the reactions theoretically lead to only a unique thermodynamically stable phase under a specific condition. Moreover, deriving the jump frequency of less mobile ions from the calculated migration energy allows the possibility of predicting the temperature at which the metastable phase becomes kinetically unstable. Thus, calculating migration barriers for each atom in computationally predicted materials would enable evaluation of their kinetic synthesizability, thereby facilitating predictive synthesis for more expedient exploration of kinetically stabilized metastable materials.

## 4 Experimental Section

4.1

4.1.1

##### Experimental

All experiments were conducted within a glovebox filled with Ar at a dew point of ≈−80 °C. LiCl (Kojundo Chemical Lab.) and LiBr (Sigma‐Aldrich) were purchased and used as reagents after grinding with a planetary ball mill (Fritsch P‐7). YCl_3_ (Sigma‐Aldrich) and YBr_3_ (Wako) were used as received without any treatment. The mortars were dried overnight at 50 °C under a vacuum. In addition, LiCl, YCl_3_, LiBr, and YBr_3_ were mixed and sealed in a quartz glass capillary under a vacuum. Synchrotron X‐ray diffraction patterns were measured at the BL02B2 beam line of Spring‐8 (Proposal Nos. 2019B1195, 2020A1096). The wavelength of the incident X‐rays were 0.496391 or 0.495395 Å. The sample capillaries were heated at a rate of 30 K min^−1^. Diffraction data were collected using a high‐resolution one‐dimensional semiconductor detector.^[^ ^44^ ^]^ The X‐ray diffraction data were analyzed using the Rietveld method with the RIETAN‐FP program,^[^ ^45^ ^]^ and crystal structures were drawn using the VESTA program.^[^ ^46^ ^]^ An *β*‐Li_3_YCl_6_ powder sample was synthesized by heating a cold‐pressed pellet of 3LiCl+YCl_3_ at 595 K for over 50 h. The time‐of‐flight powder neutron diffraction data of *β*‐Li_3_YCl_6_ were collected on the SPICA diffractometer installed at the Material and Life Science Facility in the Japan Proton Accelerator Research Complex (Proposal No. 2017L1302). The neutron diffraction pattern obtained with the low‐angle bank and backscattering bank was refined using the Z‐Rietveld program.^[^ ^47^ ^]^ In this paper, results for backscattering bank was shown in main body since resolution with backscattering bank is higher than that of low angle bank. The ionic conductivity of pelletized *β*‐Li_3_YCl_6_ was determined using electrochemical impedance spectroscopy. The sample powder was pressed into a pellet with a diameter of 6 mm, and two stainless steel current collectors were used. Electrochemical impedance spectroscopy (ESI) was measured in an Ar‐filled glovebox under ≈280 MPa using an impedance analyzer (Sl1260) within the frequency range of 0.1–10^7^ Hz at a voltage amplitude of 30 mV. During the ESI measurement, the dew point of the Ar‐filled glovebox was below −70 °C. Thermogravimetric (TG)‐DSC measurement was performed by NEXTA STA300 in a N_2_ flow. Scanning electron microscopy (SEM) and transmission electron microscopy (TEM) observations were performed by JEM‐2010 and JIB‐4600F, respectively, without exposing to air during sample transfer.

##### Computational

First‐principles calculations based on the density functional theory were conducted to investigate the structural and migration energies using the projector augmented‐wave (PAW) method implemented in the VASP code.^[^ ^48^, ^49^, ^50^, ^51^ ^]^ For the calculation of *β*‐Li_3_Y*X*_6_ (*X*: Cl and Br), the crystal structure model shown in Figure S10a (Supporting Information) was considered. To estimate the energy of *β*‐Li_3_Y*X*_6_, a supercell containing 2 × 2 × 2 unit cells was constructed with random arrangements of Li and Y sublattices based on occupancy. The crystal structure model shown in Figure S10b (Supporting Information) was also considered for the calculation of α‐Li_3_Y*X*_6_. A supercell containing 2 × 1 × 1 unit cells with a random arrangement of Li was constructed to estimate the energy of α‐Li_3_Y*X*_6_. The average energies of 100 structures with random arrangements were considered as the total energies of the *β*‐ and *α*‐Li_3_Y*X*_6_ structures. The exchange‐correlation term was treated with GGA‐PBE.^[^ ^52^ ^]^ The plane‐wave cutoff energy was set to 350 eV. The integration in the reciprocal space was conducted using 2 × 2 × 2 and 3 × 3 × 2 Γ‐centered grids for the *β*‐ and *α*‐Li_3_Y*X*_6_, respectively. Structure optimization was carried out until all residual forces acting on each atom became less than 0.02 eV Å^−1^.

Next, the migration energies of the atoms were estimated by assuming a vacancy mechanism. To calculate the migration energies of atoms in the *β*‐Li_3_Y*X*_6_, the initial and final state models of the crystal structure need to be defined. All symmetrically independent atomic configurations in the *β*‐Li_3_Y*X*_6_ unit cell were calculated with two configurations, as shown in Figure S11 (Supporting Information). The atomic configuration shown in Figure S11a (Supporting Information) has a lower energy than that shown in Figure S11b (Supporting Information) by 0.04 and 0.14 eV for *X* = Cl and Br, respectively. Thus, a structure with the atomic configuration shown in Figure S11a (Supporting Information) was considered as the initial model. For Li migration, the paths from the first to the third nearest neighbor Li atoms to the Li vacancies was calculated. The nearest‐neighbor Y atoms from the Y vacancy were considered for Y migration. However, there were no *X* vacancies in the structure. Thus, the crystal structure with a point defect of V*_X_* ^•^ was first calculated. The *X* atom migrations from the nearest neighbor sites were also calculated. Note that the total number of calculated migration paths for *X* is 12 (the number of nearest neighbor sites), because these sites became symmetrically independent owing to the addition of the *X* vacancy. The migration energies for each atom in *β*‐Li_3_Y*X*_6_ were calculated using the climbing image nudged elastic band method with one intermediate image.^[^ ^16^ ^]^ Only the internal parameters were optimized.

## Conflict of Interest

The authors declare no conflict of interest.

## Supporting information

Supporting Information

## Data Availability

The data that support the findings of this study are available from the corresponding author upon reasonable request.
