# Supplementary material for: Kinetically Stabilized Cation Arrangement in Li3YCl6 Superionic Conductor during Solid‐State Reaction
Source: Adv Sci (Weinh). 2021 Jun 17;8(15):2101413. doi: 10.1002/advs.202101413 (PMC8336504; doi:10.1002/advs.202101413)
Supplement: Supplementary file 1 — Supporting Information [file ADVS-8-2101413-s001.pdf]

© 2021 Wiley-VCH GmbH

ADVANCED SCIENCE
Open Access

Supporting Information

for *Adv. Sci.*, DOI: 10.1002/advs.202101413

Kinetically Stabilized Cation Arrangement in Li_3YCl_6 Superionic Conductor during Solid-State Reaction

Hiroaki Ito,[a] Kazuki Shitara,[b,c] Yongming Wang,[d] Kotaro Fujii,[e] Masatomo Yashima,[e] Yosuke Goto,[f] Chikako Moriyoshi,[g] Nataly Carolina Rosero-Navarro,[h] Akira Miura,[h] Kiyoharu Tadanaga[h]*

Supporting Document: Kinetically Stabilized Cation Arrangement in Li_3YCl_6 during Solid-State Reaction

Hiroaki Ito,[a] *Kazuki Shitara,*[b c] *Yongming Wang,*[d] *Kotaro Fujii,*[e] *Masatomo Yashima,*[e] *Yosuke Goto,*[f] *Chikako Moriyoshi,*[g] *Nataly Carolina Rosero-Navarro,*[h] *Akira Miura,* [h]* *Kiyoharu Tadanaga* [h]

Corresponding author: Akira Miura amiura@eng.hokudai.ac.jp

[a]Graduate School of Chemical Science and Engineering, Hokkaido University, Kita 13, Nishi 8, Sapporo, Hokkaido 060-8628, Japan.

[b]Joint and Welding Research Institute, Osaka University, 11-1 Mihogaoka, Ibaraki, Osaka, 567-0047, Japan

[c]Nanostructures Research Laboratory, Japan Fine Ceramics Center, 2-4-1, Mutsuno, Atuta, Nagoya, Aichi, 456-8587, Japan

[d]Creative Research Institution Hokkaido University, Kita 21, Nishi 10, Sapporo, Hokkaido, 001-0021, Japan

[e]Department of Chemistry, School of Science, Tokyo Institute of Technology, 2–12–1 W4–17 O-okayama, Meguro, Tokyo, 152–8551, Japan

[f]Department of Physics, Tokyo Metropolitan University, 1-1 Minami-Osawa, Hachioji, Tokyo, 192-0397, Japan.

[g]Graduate School of Advanced Science and Engineering, Hiroshima University, 1-3-1 Kagamiyama, Higashihiroshima, Hiroshima, 739-8526, Japan

[h]Faculty of Engineering, Hokkaido University, Kita 13, Nishi 8, Sapporo, Hokkaido, 060-8628, Japan.

Rietveld Refinement Detail

The crystal structure, proposed in Figure 2, was solved in three steps. First, Rietveld refinement of SXRD was conducted by optimizing the lattice parameters, occupancies, displacement parameters of Y (2*b*) and Cl (6*f*). First, the occupancy and the displacement parameter of Cl (6*f*) were fixed, those of Y (2*b*) were refined. Second, those of both Y (2*b*) and Cl (6*f*) were refined. As these occupancies converged at 0.5 and 1 within the error, we applied further refinements with these fixed occupancies. Final refinement was performed with fixed atomic occupancy and displacement parameters determined by ND.

We applied an ND diffraction to refine those of Li (4*d*). There are two ND settings. One is a low-angle bank showing a wide-angle range, but the resolution is moderate. The other is a backscattering bank giving only a low-angle range, but the resolution is high. Accordingly, the reported crystal structure shown in Table 1 is about the ND measurement at the backscattering bank while the diffraction data of the low angle bank shows no diffraction peaks corresponding to the superlattice of the proposed lattice. For the ND refinements, the first refinement was performed with the isotropic atomic displacement parameter of Li(4*d*), and further refinements were performed with anisotropic atomic displacement parameters for Li (U_{11}(Li) and U_{33}(Li)). Although the anisotropic displacement parameters for Y and Cl had been refined, these atomic displacement parameters were isotropic within error. Thus, Y and Cl were refined using isotropic displacement parameters. Furthermore, each refinement of occupancies of Li(4*d*), Y (2*b*), and Cl (6*f*) did not converge with better refinement parameters.

The phase fraction shown in Figure 1 was derived from Rietveld refinement of the *in situ* SXRD. All atomic information of Li was fixed, and only the lattice parameters, atomic position, and isotropic displacement parameters of Y and Cl were refined. We applied refinements using the α phase, which can represent the β phase by changing the occupation of Y. Thus, the phase fraction of the α/β phase was derived from the occupancies of the Y sites.

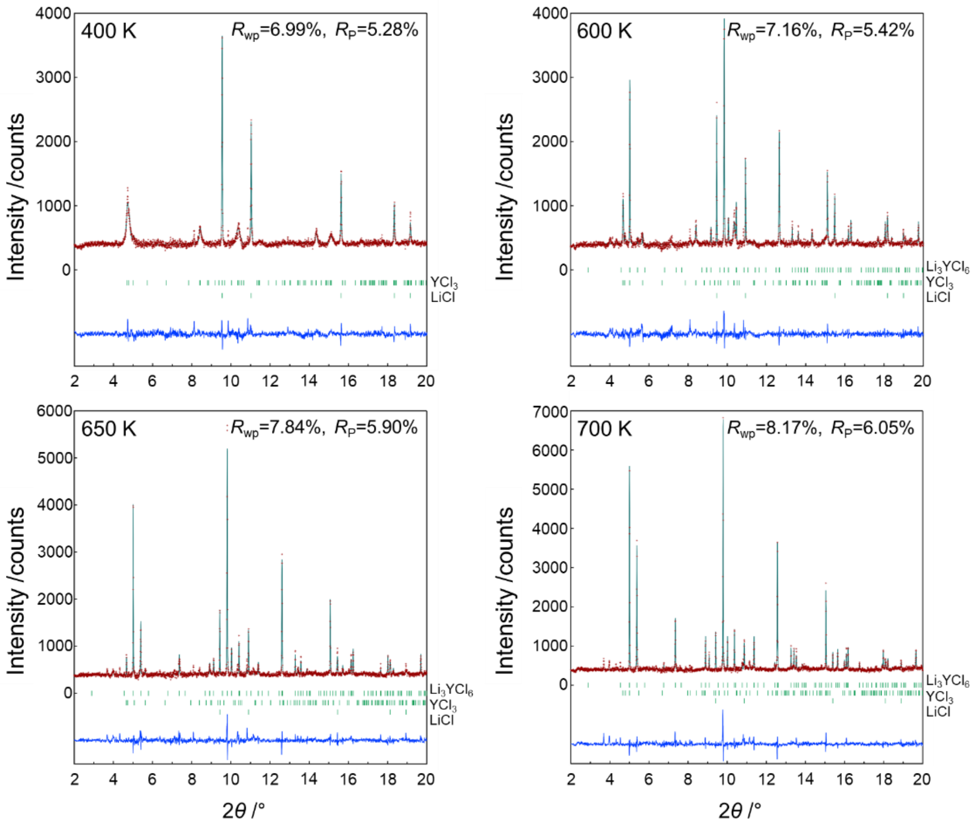

Figure S1 Rietveld profiles of *in situ* XRD patterns for LiCl and YCl_3 measured at 400, 600, 650, and 700 K. The experimental and calculated results are indicated by red dots and green solid lines, respectively, in each graph. The bottom blue lines are the residuals.

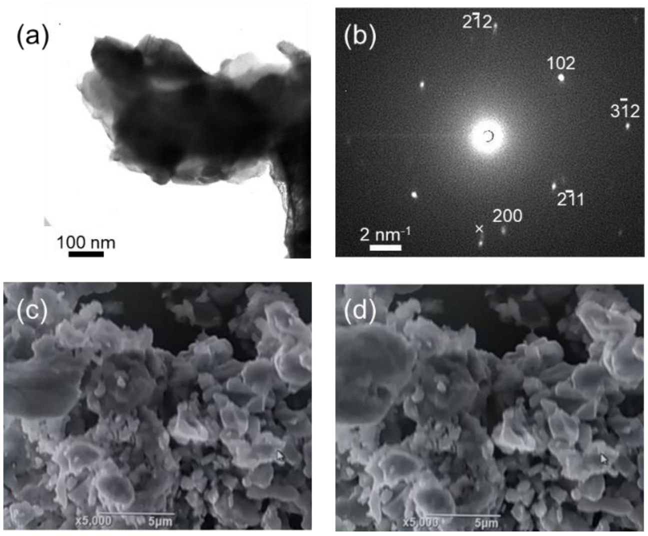

Figure S2 Electron microscope image of β-Li_3YCl_6. (a) TEM and (b) corresponding electron diffraction images of β-Li_3YCl_6 synthesized by heating at 595 K for 50 h. The numbers are indexes of β-Li_3YCl_6, and a weak spot shown as X cannot be indexed as β-Li_3YCl_6. SEM images of the sample (c) after ~10 sec and (d) after ~60 sec. The change in morphology indicates an unstable feature under the electron beam.

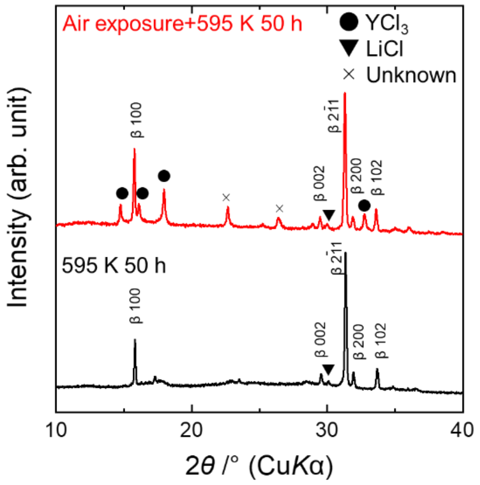

Figure S3 *ex-situ* XRD patterns of Li_3YCl_6 synthesized at 595 K with/without exposure to Air. Precursor powders were pressed into a pellet with a diameter of 6mm and exposed to air for ~1 minute before sealed in an evacuated quartz tube.

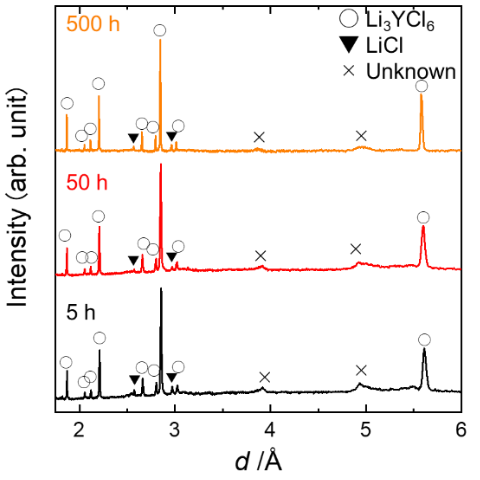

Figure S4 Time dependence of *ex-situ* XRD patterns of Li_3YCl_6 heated at 595 K for 5-500 h.

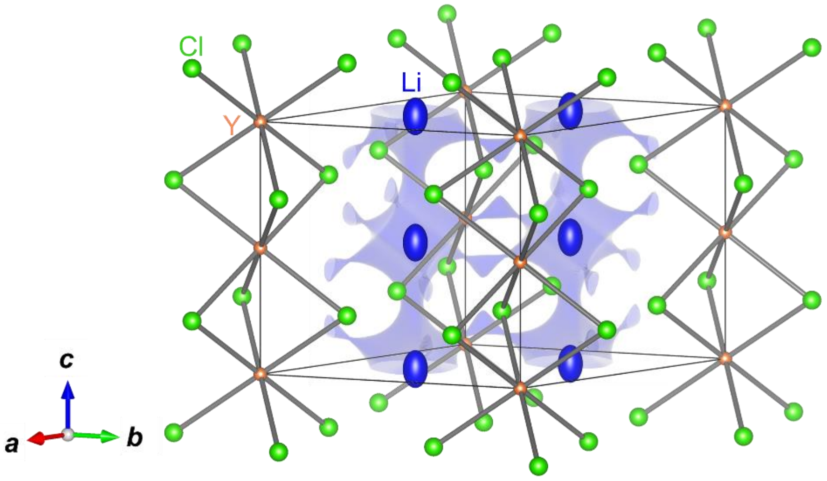

Figure S5 Oak ridge thermal ellipsoid plot drawing (75% thermal ellipsoids) and bond valence sum (BVS) mapping for Li_3YCl_6. Blue, orange, and green ellipsoids represent lithium, yttrium, and choline, respectively. The isosurface is drawn in transparent blue at a BVS mismatch level of 0.35 valence units for Li^+.

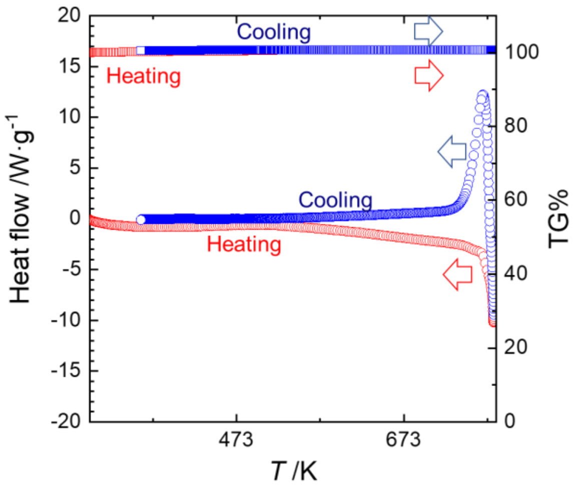

Figure S6 TG/DSC of β-Li_3YCl_6 synthesized at 595 K for 50 h measured at the temperature sweep speed of 30 K/min under N_2 flow.

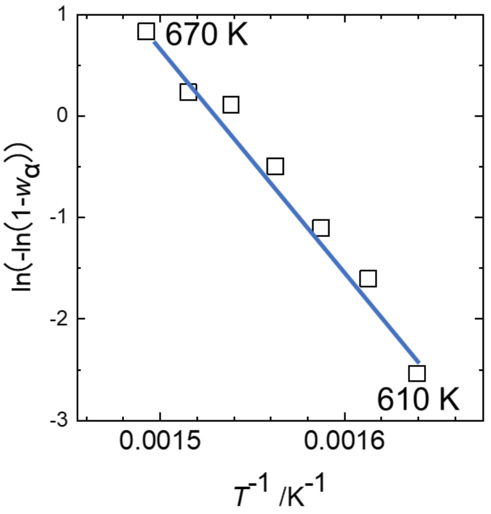

Figure S7 Activation energies derived from the temperature dependence of the phase fraction. w_α is the mass fraction of α-phase over the sum of β and α-phases. The Avrami constant was estimated to be 1 assuming Y migration along the c-axis shown in Figure 4.

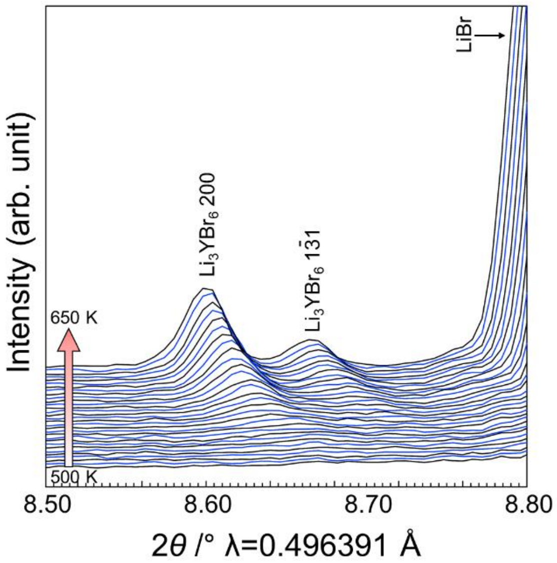

Figure S8 Temperature dependence of in-situ XRD of the mixture of LiBr and YBr_3.

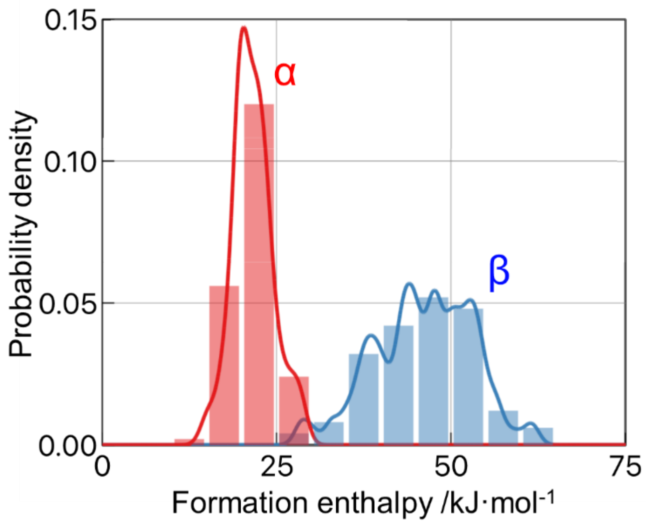

Figure S9 Calculated configurational density of states of Li_3YBr_6 isostructural with α- or β-Li_3Y Br_6 by sampling 100 structures.

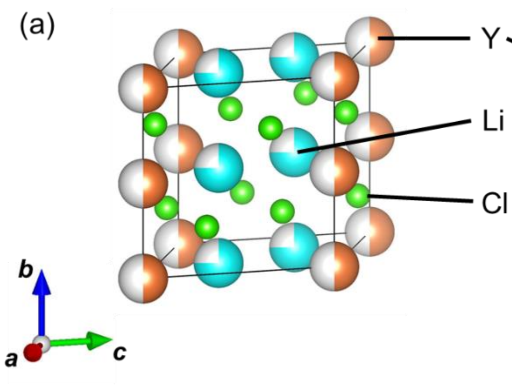

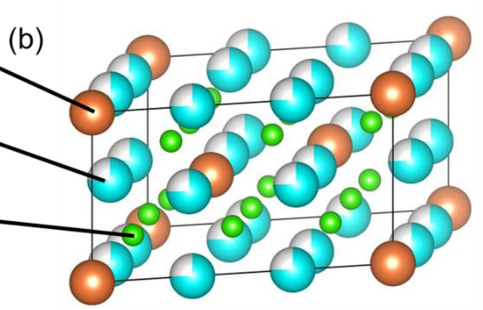

	occupancy	*x*	*y*	*z*
Y	0.5	0	0	0
Li	0.75	1/3	2/3	0
Cl	1	1/3	0	0.25

	occupancy	*x*	*y*	*z*
Y1	1	0	0	0
Y2	1	1/3	2/3	0.5119
Li1	0.75	0.3333	0	0
Li2	0.75	0.322	0.0	0.5
Cl1	1	0.1144	-0.1144	0.7706
Cl2	1	0.2213	-0.2213	0.2643
Cl3	1	0.4469	-0.4469	0.7536

Figure S10 Structure models of supercells employed for first-principles calculations. (a) β-Li_3YCl_6 (b) α-Li_3YCl_6

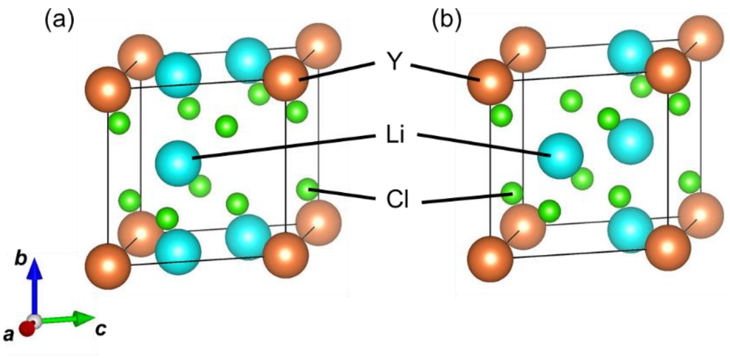

Figure S11 Crystal structure models of β-Li_3YCl_6 with different atomic configurations. (a) initial state (b) unstable state

Appendix

The ionic conductivity σ can be expressed as a product of the number of ions per unit volume, N, ionic charge, q, and mobility of the particles, μ:

$\sigma = Nq\mu$ eq. (3)

From the Nernst-Einstein equation, μ can be also given by:

$\mu = \frac{Dq}{k_B T}$ eq. (4)

The influence of temperature on the diffusion constant D is represented by the Arrhenius equation.

$D = D_0 \cdot \exp\frac{-E_a}{RT}$ eq. (5)

From eq. (3), (4), and (5), σ is given by:

$\ln\sigma = -\frac{E_a}{R}\left(\frac{1}{T}\right) + \ln\left(\frac{Nq^2 D_0}{k_B T}\right)$ eq. (6)

Table S1 Synthesis condition, lattice parameters, and ionic conductivity of Li_3YCl_6.

	Precursor, molar ratio	Heating condition	Lattice parameter [Å]		Ionic conductivity at 25 ℃ [S·cm^{-1}]
			a	*c*	
β-Li_3YCl_6 (this paper)	LiCl: YCl_3=3:1	595 K for 50 h	6.4604(1)	6.0302(1)	1.2×10^{-4}
α-Li_3YCl_6 (this paper)	LiCl: YCl_3=3:1	700 K for 0.5 h	11.1848(1)	6.02606(9)	1.4×10^{-5}
Ball-milled Li_3YCl_6 (1)	LiCl: YCl_3=3:1	Only ball milling	11.2217(7)	6.05517(6)	5.1×10^{-4}
α-Li_3YCl_6 (2)	LiCl: YCl_3=3:1	Annealing at 823 K	11.1969(0)	6.03112(3)	~3.0×10^{-5}
α-Li_3YCl_6 (3)	LiCl: YCl_3=3:1.1	823 K for 1 week + slow cooling	11.20934(9)	6.04694(9)	~6.1×10^{-5}
α-Li_3YCl_6 (4)	LiCl: YCl_3=3:1.1	823 K for 5 min + air quenching	11.20926(9)	6.0382(1)	~3.7×10^{-5}

(1, 2) T. Asano *et al*., Solid halide electrolytes with high lithium-ion conductivity for application in 4 V class bulk-type all-solid-state batteries, *Advanced Materials* **30**, 1803075 (2018).

(3, 4) R. Schlem *et al*., Insights into the lithium substructure of the superionic conductors Li_3YCl_6 and Li_3YBr_6, *Chemistry of Materials* **33**, 327-337 (2021)

Note: The melting point of Li_3YCl_6 is 765 K, [Y, Sun *et al*., Thermodynamic optimization and calculation of the YCl_3-ACl (A=Li, Na, K, Rb, Cs) phase diagrams, *CALPHAD: computer coupling of phase diagrams and thermochemistry* **39**, 1-10 (2012).] Thus, heating at 823 K likely brought about a compositional change due to highly reactive LiCl with a quartz tube [M. Nagao Crystal Growth Techniques for Layered Superconductors. *Condensed Matter* **2**, 32 (2017)

Table S2-1 Fractional coordinates, occupancies, atomic displacement parameters, and bond valence sum of β-Li_3YCl_6 refined using synchrotron X-ray diffraction. Anisotropic atomic displacement parameters for Li were fixed, and isotropic displacement of Y and Cl were refined. Lattice Parameters: a= 6.4604 (1) Å, c=6.0302(1) Å, R_{wp}=5.16%, R_p=3.63%, R_R=50.34%, R_e=2.58%, S=2.0009, Space group:165 $P\bar{3}c1$, The second phase: 9.32 mass% of LiCl

Atom label	Wyckoff position	Occupancy	Atomic coordinates			U [Å^2]	BVS
			x	y	z		
Y	2*b*	0.5	0	0	0	0.008(1)	3.234
Li	4*d*	0.75	1/3	2/3	0	0.013 (fix)	0.86
Cl	6*f*	1	1/3	0	1/4	0.017(1)	0.969

* U_{11}(Li) =U_{22}(Li) = 0.03710 Å^2, U_{33}(Li)= 0.0858 Å^2

Table S2-2 Fractional coordinates, occupancies, and atomic displacement parameters of β-Li_3YCl_6 refined using Neutron diffraction collected at low angle bank. Isotropic atomic displacement parameters for Y and Cl were fixed, and anisotropic atomic displacement parameters, U_{11} and U_{33}, were employed only for Li. Lattice Parameters: a= 6.47636(2) Å, c=6.05680(4) Å, R_{wp}=9.51, R_P=5.51%, R_e=0.45%, R_B(Li_3YCl_6)=10.18%, R_F(Li_3YCl_6)=3.72%, Space group:165 $P\bar{3}c1$, The second phase: 2.6 mass% of LiCl

Atom label	Wyckoff position	Occupancy	Atomic coordinates			U [Å^2]	BVS
			x	*y*	*z*		
Y	2*b*	0.5	0	0	0	0.0110(3)	3.163
Li	4*d*	0.75	1/3	2/3	0	0.0649(7)	0.841
Cl	6*f*	1	1/3	0	1/4	0.0206(1)	0.948

* U_{11}(Li) =U_{22}(Li) = 0.058(1) Å^2, U_{33}(Li)= 0.079(1) Å^2
